# Stress-Dependent Optical Extinction in Low-Pressure Chemical Vapor Deposition Silicon Nitride Measured by Nanomechanical Photothermal Sensing

**DOI:** 10.1021/acs.nanolett.4c02902

**Published:** 2024-08-30

**Authors:** Kostas Kanellopulos, Robert G. West, Stefan Emminger, Paolo Martini, Markus Sauer, Annette Foelske, Silvan Schmid

**Affiliations:** † Institute of Sensor and Actuator Systems, TU Wien, 1040 Vienna, Austria; ‡ Analytical Instrumentation Center, TU Wien, 1060 Vienna, Austria

**Keywords:** absorption, extinction, nanomechanics, nanophotonics, optics, photonics

## Abstract

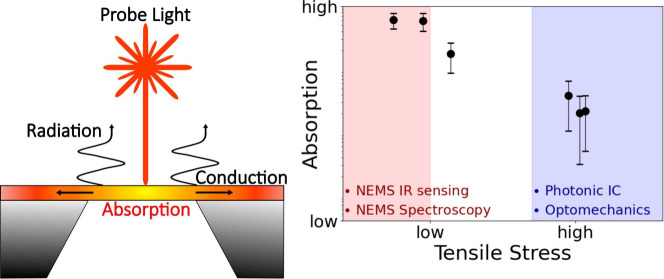

Understanding optical absorption in silicon nitride is crucial for cutting-edge technologies like photonic integrated circuits, nanomechanical photothermal infrared sensing and spectroscopy, and cavity optomechanics. Yet, the origin of its strong dependence on the film deposition and fabrication process is not fully understood. This Letter leverages nanomechanical photothermal sensing to investigate optical extinction κ_ext_ at a 632.8 nm wavelength in low-pressure chemical vapor deposition (LPCVD) SiN strings across a wide range of deposition-related tensile stresses (200–850 MPa). Measurements reveal a reduction in κ_ext_ from 10^3^ to 10^1^ ppm with increasing stress, correlated to variations in Si/N content ratio. Within the band-fluctuations framework, this trend indicates an increase of the energy bandgap with the stress, ultimately reducing absorption. Overall, this study showcases the power and simplicity of nanomechanical photothermal sensing for low absorption measurements, offering a sensitive, scattering-free platform for material analysis in nanophotonics and nanomechanics.

Investigating the optical properties of solid-state materials is essential for both fundamental and applied science. Optical absorption, in particular, is critical in various fields, including photonic integrated circuits (PIC) for quantum information,^1^ and the design of nanomechanical resonant sensors for infrared (IR) light detection,^2^ photothermal spectromicroscopy,^3−5^ and cavity optomechanics.^6−10^

In IR sensing, high optical absorption is desired to enhance the sensor’s specific detectivity.^2,11^ Conversely, applications like PICs, cavity optomechanics, and nanomechanical photothermal spectromicroscopy require minimal absorption to realize high-confinement waveguides,^12^ to prevent mechanical instability^10,13^ and cavity bistability,^14^ and to mitigate photothermal back-action frequency noise introduced in the resonator,^15^ respectively.

Silicon nitride (SiN) holds a prominent position in these fields for its excellent mechanical, thermal, and optical properties.^16,17^ Its extensive use in photonics stems from its broad transparency window (0.4–8 μm), which, however, strongly depends on the film deposition and fabrication process, for which the underlying mechanisms are not fully understood. This has been observed by means of various characterization techniques, such as ellipsometry,^18−23^ direct single-pass absorption spectroscopy,^24^ FTIR interferometry,^25,26^ cutback,^27^ outscattered light method,^28−32^ prism coupling,^33^ photoluminescence,^34^ photothermal common-path interferometry,^35^ and cavity-enhanced absorption spectroscopy.^12,16,36^ However, these approaches often suffer from scattering losses and slow measurement time, which obscure the true absorption of SiN and make analyses prone to parasitic heating of the surroundings.

Within this context, nanomechanical photothermal spectroscopy offers a robust solution to these challenges.^4,37−42^ Here, a tensile stressed nanomechanical resonator detects directly absorption via resonance frequency shifts due to photothermal heating, insensitive to scattering. Upon illumination, the resultant temperature rise makes the initial tensile stress relax, leading to frequency detuning. Due to its high power sensitivity, fast thermal response, and versatility in sensor design, this technique has significantly advanced the characterization of low-loss materials.^5^

In this Letter, nanomechanical photothermal sensing is employed to elucidate the relationship between absorption and residual tensile stress in low-pressure chemical vapor deposition (LPCVD) deposited SiN thin films. The extinction coefficient at 632.8 nm wavelength is measured from low stress (≈200 MPa), relevant to photothermal-based applications,^5^ to high stress (>800 MPa), relevant to cavity optomechanics^43,44^ and PIC design.^12^ The thin films are patterned in a string geometry, which ensures high photothermal responsivity and fast response, as previously demonstrated.^15^ The experimental results reveal a reduction in extinction (from 10^3^ to 10^1^ ppm) with increasingly higher tensile stress, consistent with previously observed trends.^23^ The measurements are analyzed within the framework of the band-fluctuations model,^45^ attributing the observed reduction to a blue-shift in the energy bandgap caused by a decrease in silicon-to-nitrogen (Si/N) content ratio. Overall, this study underscores the power and simplicity of nanomechanical photothermal sensing for the characterization of low-loss materials in nanophotonics and nanomechanics.

In the present setup, the nanomechanical resonator is operated in a custom-made vacuum chamber at high-vacuum conditions (*p* < 10^–5^ mbar) to reduce gas damping and thermal convection losses.^4^ The mechanical displacement is transduced optically via laser-Doppler vibrometry (LDV, Polytec GmbH MSA-500) equipped with a HeNe laser at 632.8 nm wavelength, with a beam waist of ≈1.5 μ m (Figure 1). The same laser also probes SiN absorption, which simplifies the measurement procedure. For each structure, the frequency shift of the thermomechanical noise peak for the fundamental resonance mode is recorded at various optical powers (6–120 μW),^15,46^ as schematically shown in Figure 1b. Here, the plot shows the displacement power spectral density (PSD), in units [m^2^/Hz], around the fundamental mechanical resonance. Its peak *S*_zz,thm_, well resolved by the vibrometer (*S*_zz,thm_ ≫ *S*_det_, with *S*_det_ denoting the detection noise PSD), red-shifts as the impinging optical power increases (*f*_*i*_ > *f*_*i*+1_ for *P*_*i*_ < *P*_*i*+1_). For each experimental result, the system has been measured in the steady state, recording the signal for a measurement time τ_meas_ > τ_th_, with τ_th_ denoting the time required for the resonator to reach this steady state (see eq 1).^15^ A minimum of five resonators is evaluated for each stress and length, with fundamental resonance frequencies ranging from 60 kHz (for *L* = 2 mm and σ_0_ = 174 MPa) to 2.6 MHz (for *L* = 0.1 mm and σ_0_ = 835 MPa). Figure 1c shows a representative image of the characterized strings.

**Figure 1:**
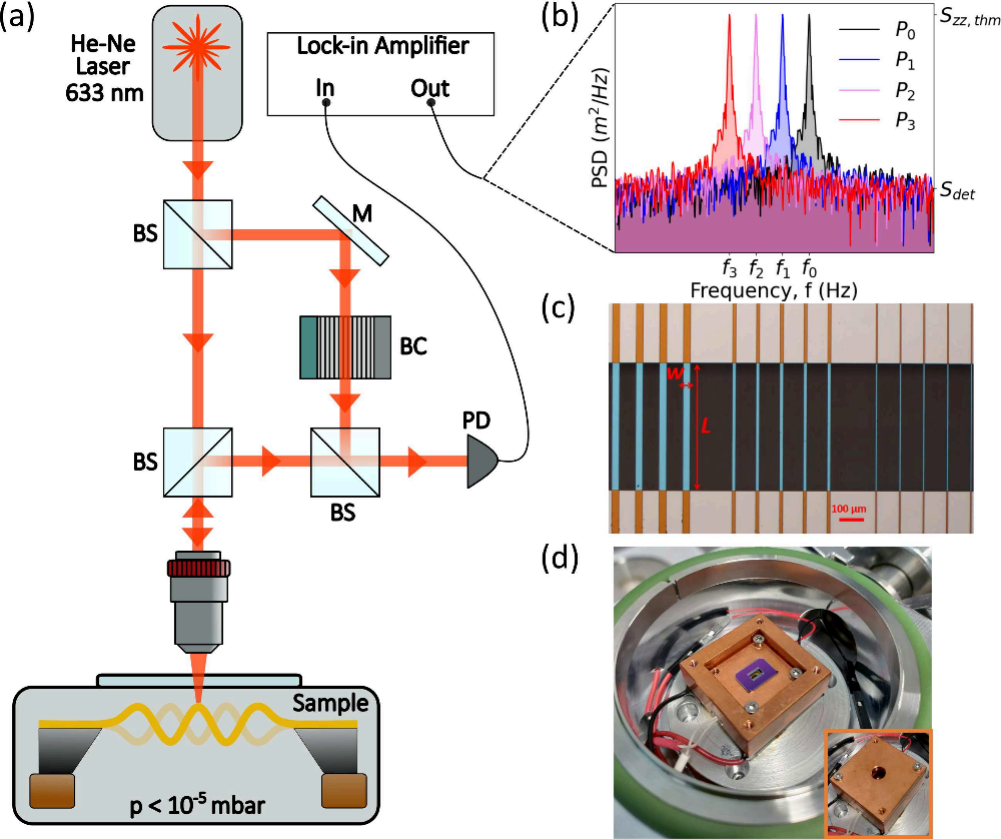
(a) Sketch of the experimental setup (LDV MSA-500, Polytech GmbH). A laser of wavelength λ and input power *P*_0_ impinges on the resonator, of absorption coefficient α_abs_(λ). This causes a frequency detuning of the nanomechanical resonator. BS: beam splitter. BC: Bragg cell. PD: photodetector. (b) Mechanical frequency detuning measured by monitoring the shift of the thermomechanical noise peak of the string’s fundamental mode as a function of *P*_0_. The peak *S*_zz,thm_ is given in terms of displacement power spectral density (PSD) [m^2^/Hz] and is well resolved by the vibrometer (*S*_zz,thm_ ≫ *S*_det_, with *S*_det_ denoting the detection noise PSD). In the *x*-axis, *f*_*i*_ = *f*_res_(*P*_*i*_), with *i* = 1, 2, ···, is the resonance frequency of the fundamental mode at each input laser power *P*_*i*_ > *P*_*i*–1_. (c) Optical micrograph of the SiN strings used in the present study. Orange/light blue regions are made of SiN; the gray regions are the Si substrate. (d) Photo of the cm-scale copper thermal equilibrium chamber used for the characterization of the linear coefficient of thermal expansion. A thermoelectric module is glued beneath to heat the whole oven (thick red electrical connections) to guarantee a uniform temperature rise of the chips. The temperature is monitored and kept constant with a PID controller.

The absorption coefficient α_abs_ is determined via comparison between the theoretical  and the experimental  relative power responsivity for the string resonators.^5,15,47^ On the one hand,  denotes the relative frequency change per absorbed power *P*(λ) = α_abs_(λ)*P*_0_ and is expressed as^47^
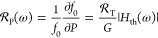
1where , *G*, and *H*_th_(ω) = (1 + i*ωτ*_th_)^−1^ denote the temperature responsivity in units [1/K], the thermal conductance in units [W/K], and the thermal response of the resonator, with τ_th_ its thermal time constant, respectively.^15,47^ As already mentioned, all the measurements have been performed far from any thermal transient (ω ≪ τ_th_^–1^), i.e., in the steady-state. Hence, the ω-dependence is dropped in the following.

For a string resonator, eq 1 is given by^47^
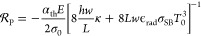
2with α_th_, *E*, σ_0_, *h*, *w*, *L*, κ, ϵ_rad_, σ_SB_, and *T*_0_ denoting the resonator’s linear coefficient of thermal expansion, Young’s modulus, tensile stress, thickness, width, length, thermal conductivity, emissivity, Stefan–Boltzmann constant, and bath temperature, respectively. The thermal conductance *G* includes the thermal dissipation through the surrounding frame *G*_cond_ (first addend in brackets) and thermal radiation to the environment *G*_rad_ (second addend in brackets).^15,47^

 is obtained by directly measuring the relative frequency shift per impinging power *P*_0_ (as schematically shown in Figure 1b). It relates to  through the absorption coefficient α_abs_(λ) as follows
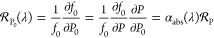
3with λ denoting the probe optical wavelength. From eq 3, it is possible to directly evaluate the optical absorption coefficient as .

The experimental power responsivity (3) across the different stresses is displayed in Figure 2a as a function of the resonators’ length *L* (circles), together with the theoretical calculations (2) (solid curves). The scale is given in terms of absorbed power *P*. It is worth noting that  grows for longer strings in the conduction-limited regime (*L* < 1 mm), as *G* ≃ *G*_cond_ is inversely proportional to the length *L* (see eq 2), leading to better thermal insulation from the environment.^15^ Conversely, increasingly longer resonators (*L* > 1 mm) enter the radiation-limited regime (*G* ≃ *G*_rad_), leading to a drop of  as the radiating surface increases.

**Figure 2:**
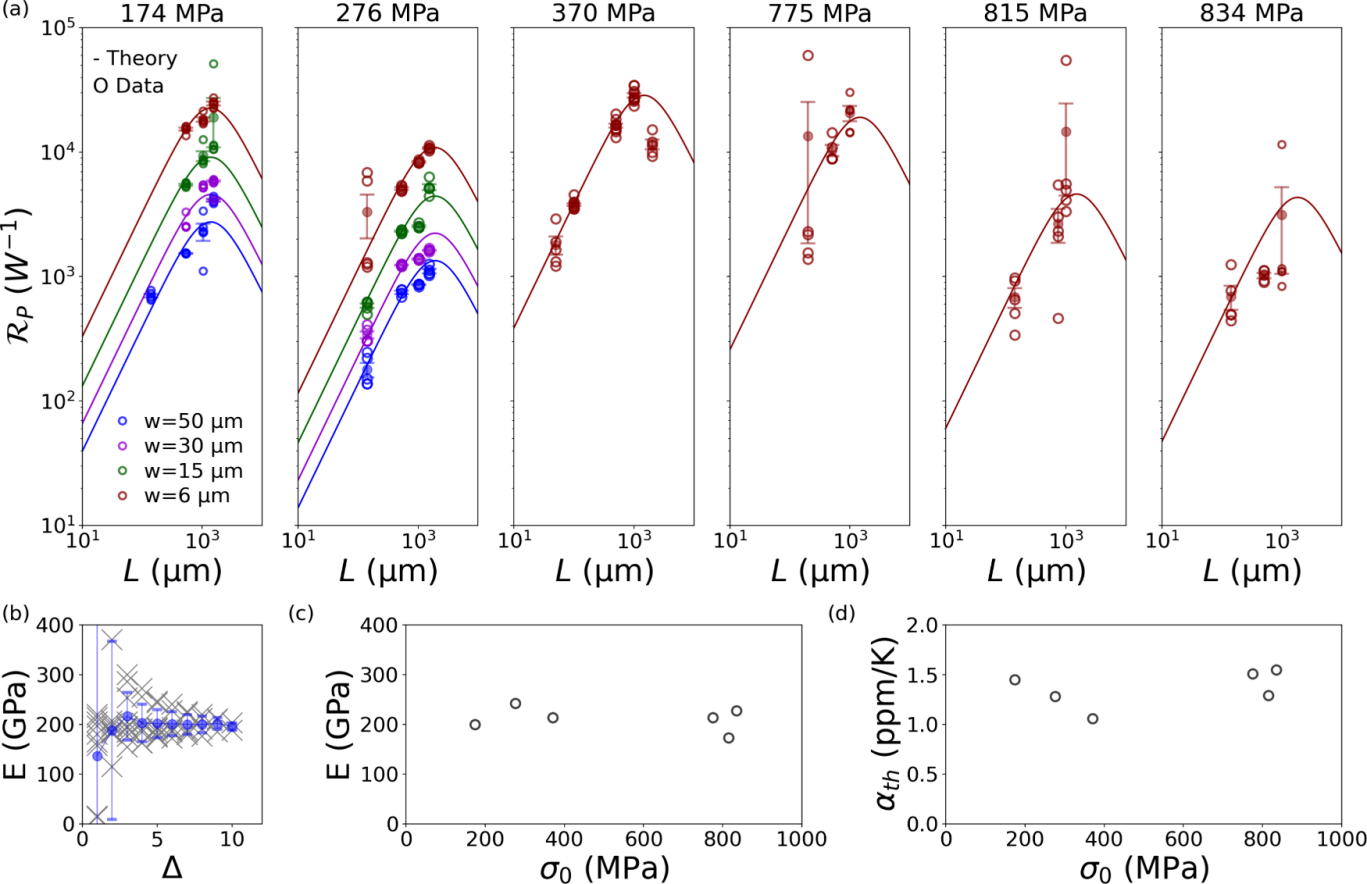
(a)  for different SiN string structures. Circles: experimental responsivity (3), divided by the corresponding mean absorption coefficient α_abs_. Solid curve: theoretical model (2). Material parameter assumed: ρ = 3000 kg/m^3^, κ = 3 W/(m K). Emissivity values are calculated from data reported in ref (25): 0.05 (*h* = 56 nm), 0.13 (*h* = 157 nm), 0.133 (*h* = 177 nm), 0.171 (*h* = 312 nm), 0.176 (*h* = 340 nm). (b) Example of Young’s modulus estimation, following the procedure of ref (48). (c) Experimental Young’s modulus *E* as a function of the prestress σ_0_. (d) Experimental linear coefficient of thermal expansion α_th_ as a function of the prestress σ_0_.

The analyzed resonators have thicknesses of *h* = 56–340 nm and widths of *w* = 5–50 μm, ensuring minimal thermal dissipation. As indicated in eq 2, *G*_cond_ ∝ *hw* and *G*_rad_ ∝ *w*, making these strings highly responsive to photothermal heating. Furthermore, the length *L* varies in the range 0.1–2 mm, making the resonator’s power response mainly thermal conduction limited.^15^ The current experimental approach is, therefore, less influenced by the SiN emissivity, which, according to Kirchhoff’s law, equals the optical absorption^49^—the parameter under scrutiny in this study. Moreover, the temperature responsivity  appearing in eq 2 is independent of the Poisson’s ratio ν, opposite to what occurs in, e.g., membrane resonators,^15,47^ which further reduces the uncertainty in the absorption measurement stemming from its dependence on other material parameters. Hence, the resonators employed here make this approach highly robust for solid-state material absorption characterization.

In this regard, the Young’s modulus *E* and the linear coefficient of thermal expansion α_th_ have been measured to reduce the uncertainty on the estimation of the absorption coefficient α_abs_. *E* has been estimated following the procedure of ref (48), upon recording of the strings’ eigenmode spectrum (an example is displayed in Figure 2b, where Δ = |*m* – *n*|, with *n* ≠ *m* being modal numbers). The experimental results are displayed in Figure 2c and Table 1, with values in the range 170–250 GPa, consistent with previously reported data.^17,48^

**Table 1:** String Resonators’ Geometrical (*h*), Mechanical (*σ*_0_, *E*, *α*_th_), Compositional (Si/N), and Optical (*η*, *κ*_ext_, *E*_g_, *β*^–1^) Properties

σ_0_ (MPa)	*h* (nm)	*E* (GPa)	α_th_ (ppm/K)	η	κ_ext_ (ppm)	Si/N	*E*_g_ (eV)	β^–1^ (meV)
174	177	200	1.45	1.215	606	0.96	3.23	201
275	340	243	1.28	1.105	588	0.98	3.09	183
370	56	214	1.06	1.022	176	0.89	3.62	212
775	56	214	1.51	1.022	38	0.86	3.90	208
815	157	173	1.29	1.273	20	0.83	4.21	227
834	312	227	1.55	1.268	21	0.84	4.10	217

α_th_ has been measured by recording the frequency shift of the thermomechanical noise peak as a function of controlled temperature rises (Δ*T* = 0–10 K),^50^ through the relation . For that, a thermoelectric module (GM200–127–10–15, Adaptive Power Management) has been used to heat up the resonators, while monitoring and keeping the temperature at the desired value via a PID controller (TEC-1092, Meerstetter Engineering). The chips have been enclosed inside a cm-scale copper thermal bath to guarantee thermal equilibrium through radiative heat transfer of the string with the environment (see Figure 1d). Figure 2d and Table 1 show the corresponding results, with α_th_ lying in the range 1–1.6 ppm/K (assuming α_th,Si_ = 2.6 ppm/K), which are consistent with previously reported values.^17,50^ No stress dependence has been observed for the two material parameters.

From the absorption measurements, the extinction coefficient for these thin films can be evaluated as^51^
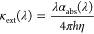
4with η denoting a dimensionless factor that accounts for possible interference inside the thin SiN slab.^42^ For the film thicknesses analyzed here, η ≈ 1–1.27 at 632.8 nm wavelength (see Table 1 and Supporting Information). Figure 3a shows the nanomechanical photothermal results of κ_ext_ as a function of the resonators’ tensile stress σ_0_ (black circles). κ_ext_ decreases from ≈10^3^ ppm for the lowest stress to ≈10^1^ ppm for the highest. These findings are compared with previously reported values of optical extinction for LPCVD (colored circles), as well as PECVD (colored diamonds), and ECR-CVD (colored squares) deposited SiN films (see Supporting Information for details on their deposition dependencies). The variance in magnitude among the compiled data for σ_0_ ≥ 850 MPa can be partially attributed also to the inability of some of the considered techniques to differentiate between true absorption and scattering losses (in particular cutback and outscattered light^12^). Overall, a general trend emerges in Figure 3a, with κ_ext_ decreasing for increasingly higher SiN deposition-related tensile stress.

**Figure 3:**
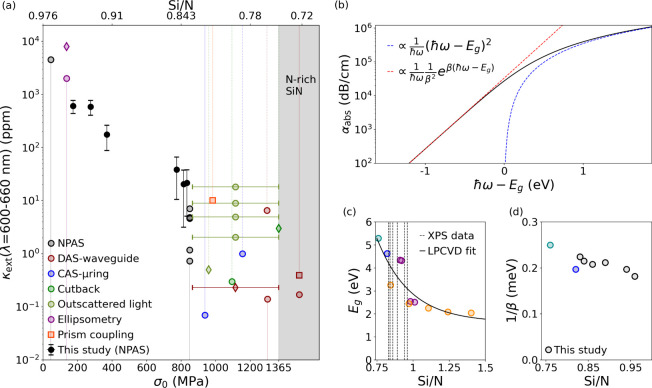
(a) κ_ext_ for different SiN string’s tensile stresses at an excitation wavelength of λ = (632.8 ± 30) nm. Characterization techniques included in the figure are: nanomechanical photothermal absorption spectroscopy (NPAS),^42^ direct absorption spectroscopy (DAS) in waveguides,^52−55^ cavity absorption spectroscopy in microring resonators (CAS-μring),^12,56^ cutback,^27^ ellipsometry,^19^ and prism coupling.^33^ Markers refer to LPCVD (circles), plasma-enhanced CVD (PECVD, diamonds), and electron-cyclotron resonance CVD (ECR-CVD, squares) deposited SiN films. For the reported values, the vertical lines indicate a relationship with stress σ_0_ (intersection with the bottom *x*-axis) or Si/N (intersection with the top *x*-axis), explicitly given in (solid lines) or derived from (dashed lines) the original article. When none of these values could be extracted, a stress error bar has been used (σ_0_ = 865–1365 MPa). (b) Absorption coefficient in the band-fluctuations model. The dashed blue and red curves represent the absorption due to electronic transition between extended states (Tauc regime) and absorption due to disorder-induced localized to extended state transitions (Urbach regime), respectively. (c) Energy bandgap *E*_g_ as a function of the Si/N ratio. The solid curve is a fitting function of the displayed reported values of the form *f*(*x*) = *ae*^*−bx*^ + *c*, with *a* = 95.94 eV, *b* = 4.356, and *c* = 1.633 eV. Only LPCDV SiN films have been considered. Compilation: dark cyan, ref (57); blue, ref (12); purple, ref (23); orange, ref (21). Dashed vertical lines indicate the Si/N ratios measured in this study with XPS. Intersections with the fitting curve are given in Table 1. (d) Corresponding Urbach energy β^–1^ of the thin films analyzed in this study (black circles). For comparison, data from ref (57) (dark cyan) and ref (12) (blues) are displayed.

The measurements are analyzed within the framework of the band-fluctuations model,^45^ which describes the absorption coefficient in units of [dB/m] as a function of the excitation energy *ℏ*ω for amorphous materials as
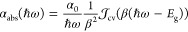
5where α_0_, β, *E*_g_, and  denote a coefficient collecting physical constants in unit [(m eV)^−1^], the Urbach slope in units [eV^–1^], the energy bandgap in units [eV], and a dimensionless joint electronic density of states (DOS), respectively. Figure 3b displays its functional form. For excitation energies *ℏω* > *E*_g_, eq 5 converges to the Tauc regime,^23^ where only fundamental electronic transitions between extended states are considered (dashed blue curve); for *ℏ*ω < *E*_g_, the model converges to the empirical Urbach tail, where electronic defect-induced absorption follows α_abs_ ∝ *e*^*βℏω*^ (dashed red curve).^45^

The model input parameters *E*_g_ and the Urbach energy β^–1^ depend on the film deposition process through the residual tensile stress present in the films. In turn, this dependence is underpinned by the underlying correlation between the stress and the corresponding Si/N ratio, with the former increasing as the latter is reduced (see Table 1), as observed in LPCVD, as well as PECVD and ECR-CVD deposited SiN films.^21,23,58^ Hence, the optical extinction reduction observed in Figure 3a for increasing tensile stress has to be related to the difference in the chemical composition of the thin films.

In this regard, the Si/N ratio of each chip has been experimentally characterized by X-ray photoelectron spectroscopy (XPS, PHI Versa Probe III-spectrometer) equipped with a monochromatic Al-K_α_ X-ray source and a hemispherical analyzer. Data analysis was performed using CASA XPS and Multipak software packages (see Supporting Information for more details). The results are displayed in Table 1 and are consistent with those reported in previous works for similar tensile stress range.^23,26,58^ These values are also shown in the top *x*-axis of Figure 3a, to highlight how SiN extinction increases with Si/N.

Finally, the energy bandgap *E*_g_ of each thin film has been extracted by means of the fitting curve constructed from the compilation of previous works on LPCVD SiN only,^12,21,23,57^ which are shown in Figure 3c. The XPS data (dashed vertical lines) are shown for clarity, and fall in the region of strongest dependence on Si/N. The corresponding energy bandgap (Table 1) has been found to increase from ≈3 eV, for the highest relative Si concentration, to ≈4.2 eV, for the lowest. All these values exceed the probing energy used in this study (*ℏω* = 1.96 eV), indicating that the absorption results from localized-to-extended electronic transitions of disorder-induced tail states, as it occurs typically in amorphous semiconductors.^45^

With the energy bandgap defined for each thin film, the corresponding Urbach energy β^–1^ has been determined by matching the experimental absorption to the band-fluctuations model. The results are shown in Figure 3d and Table 1, and are consistent with previously reported studies of LPCVD SiN (β^–1^ ≈ 200 meV).^12,57^ β^–1^ slightly decreases with increasing Si/N ratios, as it has been observed also for PECVD deposited SiN, but at lower values (see the Supporting Information for a comparison).^59,60^ Hence, lowering the Si/N ratio has the main effect of shifting the bandgap *E*_g_ to higher energies, broadening the SiN transparency window. Conversely, the Urbach energy β^–1^ does not vary significantly among these thin films, indicating that the reduction in extinction coefficient κ_ext_ is driven by an exponential decrease in the disorder-induced electronic tail DOS at the probing energy of 1.96 eV.

In conclusion, it has been shown that nanomechanical photothermal spectroscopy represents a highly sensitive, simple, and scattering-free platform for the optical characterization of low-loss materials. In this study, its capabilities have been explored using nanostring resonators made of LPCVD deposited SiN. Upon meticulous characterization of their mechanical and thermomechanical properties, it has been shown that SiN intrinsic extinction coefficient decreases with increasingly higher thin film tensile stress. This trend is attributed to a blue-shift in energy bandgap as a function of material composition. Therefore, varying the Si/N ratio provides a degree of freedom to tune the optical properties of SiN, advancing the understanding of this ubiquitous material.

## Supporting Information Available

Calculation of the emissivity and factor η for different thicknesses; compilation of data (table); derivation of Si/N ratios for the compiled data; comparison of the measured Urbach energies with reported data for LPCVD and PECVD SiN films; and details on the XPS measurements (PDF)
