# Supplementary material for: Stress-Dependent Optical Extinction in Low-Pressure Chemical Vapor Deposition Silicon Nitride Measured by Nanomechanical Photothermal Sensing
Source: Nano Lett. 2024 Aug 30;24(36):11262–8. doi: 10.1021/acs.nanolett.4c02902 (PMC11403763; doi:10.1021/acs.nanolett.4c02902)
Supplement: Supplementary file 1 — nl4c02902_si_001.pdf [file nl4c02902_si_001.pdf]

Supplementary Information: Stress-Dependent Optical Extinction in LPCVD Silicon Nitride Measured by Nanomechanical Photothermal Sensing

Kostas Kanellopulos,† Robert G. West,† Paolo Martini,† Stefan Emminger,† Markus Sauer,‡ Annette Foelske,‡ and Silvan Schmid*,†

†*Institute of Sensor and Actuator Systems, TU Wien, 1040 Vienna, Austria.*
‡*Analytical Instrumentation Center, TU Wien, 1060 Vienna, Austria*

E-mail: silvan.schmid@tuwien.ac.at

Emissivity $\epsilon_{\rm rad}$

The values of emissivity used for $\mathcal{R}_{\rm P}$ in the main text are based on the experimental data of Ref. 1. The calculations are based on the matrix formalism for a single film surrounded by vacuum,[2] equivalent to what done in Ref. 3. The results are shown in Fig. S1, with $\epsilon_{\rm rad}$ increasing with the thickness, as expected for dielectric materials.[4]

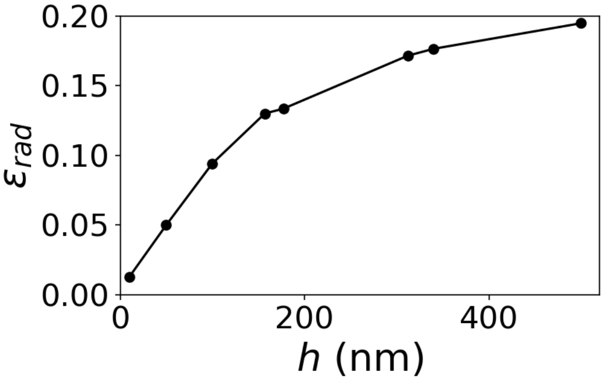

Figure S1: Emissivity $\epsilon_{\rm rad}$ as a function of the film thickness h.

Calculations of η

The factor η appearing in Eq. (4) in the main text accounts for the interference effect inside the thin film. The calculations for the values presented in the Table 1 are based on Eq. 8 of Ref. 5, equivalent to what shown in Ref. 6. For a thin film of thickness h and complex refractive index $\tilde{n} = n + i\kappa_{ext}$, probed at a wavelength λ, the absorption correction factor is given by[6]

$$\eta = \frac{4n(n^2+1) + (n^2-1)\frac{\lambda}{\pi h} sin(\frac{4\pi nh}{\lambda})}{1 + 6n^2 + n^4 - (n^2-1)^2 cos(\frac{4\pi nh}{\lambda})}. \quad \text{(S1)}$$

The dispersive part of the refractive index, n, is obtained from the Sellmeier equation[7]

$$n(\lambda)^2 = 1 + \frac{3.0249\lambda^2}{\lambda^2 - 135.3406^2} + \frac{40314\lambda^2}{\lambda^2 - 1239842^2}, \quad \text{(S2)}$$

with λ given in units of nanometer. For $\lambda = 632.8$ nm, $n = 2.04$. Fig. S2 shows how η varies with the film thickenss h at this wavelength. The values corresponding to the thicknesses used in this work are summarized in Table 1 in the main text.

Table S1: List of the extinction values and corresponding parameters used in the main text ($\lambda = 600 - 660$ nm).

Reference	Deposition	Method	λ (nm)	κ_{ext} (ppm)	σ_0 (MPa)	Si/N
6	LPCVD	NPAS	632.8	0.73<br>1.17<br>4.64<br>7.07<br>4.84	850	-
8	LPCVD<br>ECR-CVD	DAS-waveguide	630	0.17<br>0.39	-	0.73
9	PECVD	DAS-waveguide	632.8	0.23	-	-
10	LPCVD	CAS-µring	644	0.07	-	0.82
11	LPCVD	CAS-µring	632.8	1	-	0.783[a]
12	LPCVD<br>PECVD	Cutback	643	0.3<br>3	-	0.794[a]<br>0.751[b]
13	LPCVD	Outscattered light	633	18.134	-	-
14	LPCVD	Outscattered light	660	8.98	-	-
15	LPCVD	Outscattered light	630	4.91	-	-
16	LPCVD	Outscattered light	640	2.037	-	-
17	PECVD	Outscattered light	633	0.5	-	0.817[b]
18	LPCVD	DAS-waveguide	648	6.54	-	0.761[a]
19	LPCVD<br>PECVD	Ellipsometry	633	2000<br>8000	137.5	-
20	ECR-CVD	Prism coupling	632.8	10.15	-	0.813

[a] Value derived from the dispersive refractive index n_{LPCVD} for LPCVD.
[b] Value derived from the dispersive refractive index n_{PECVD} for PECVD.

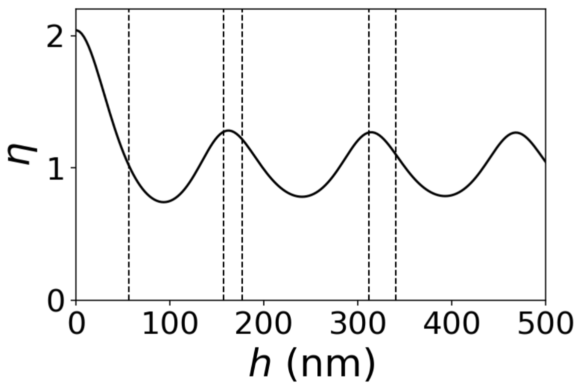

Figure S2: Factor η as a function of the film thickness h at 632.8 nm wavelength. The solid curve is calculated with Eq. (S1). The dashed vertical lines indicate the thicknesses of the films analyzed in this study.

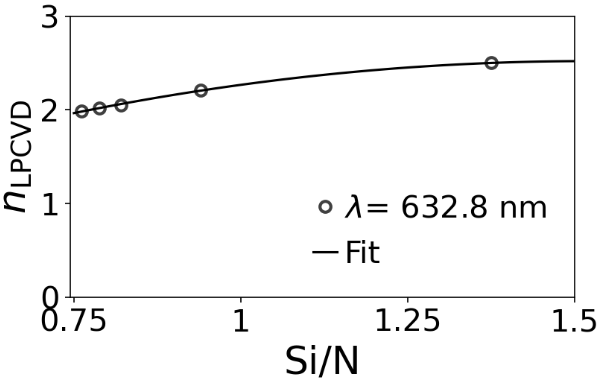

Figure S3: Refractive index as a function of the Si/N ratio in LPCVD SiN, measured at 632.8 nm wavelength (circles). The solid curve is the fit used to derive the Si/N ratio.

Data compilation

For some of the articles listed in Table S1, the derivation of the values is based on the existing relationship between the dispersive part of refractive index n and the Si/N ratio, as shown already.[21,22] The derivation is developed for each reference, where needed.

Derived parameters for LPCVD

For LPCVD SiN thin films, the measurements carried out in Ref. 21 are used to derive the Si/N ratio from n_{LPCVD} at 632.8 nm wavelength. Fig. S3 shows the reported value (circles), together with the corresponding fit. The latter has the form $f(x) = p_1x^2 + p_2x + p_3$, with $p_1 = -0.9333$, $p_2 = 2.839$, and $p_3 = 0.3608$.

Reference 11

It is reported a value of $n_{\mathrm{LPCVD}} = 2.0115$ for TE mode at 632.8 nm wavelength.

Reference 12

It is reported the following Sellmeier equation (upon fitting of the ellipsometric data)

$$n_{\mathrm{LPCVD}} = \sqrt{1 + \frac{2.926\lambda^2}{\lambda^2 - 23.47 \cdot 10^{-15}}} \qquad \text{(S3)}$$

Hence, $n_{\mathrm{LPCVD}}(643\ \mathrm{nm}) = 2.02536$.

Reference 18

It is reported a value of $n_{\mathrm{LPCVD}}(648\ \mathrm{nm}) = 1.98$.

Derived parameters for PECVD

For PECVD SiN thin film, the formula from Ref. 22 has been exploited. In particular, the following relations holds

$$\frac{\mathrm{Si}}{\mathrm{N}} = \frac{3}{4}\frac{n_{\mathrm{PECVD}} + n_{\mathrm{a-Si:H}} - 2n_{\mathrm{a-Si_3N_4}}}{n_{\mathrm{a-Si:H}} - n_{\mathrm{PECVD}}}, \qquad \text{(S4)}$$

with n_{PECVD}, $n_{\mathrm{a-Si:H}}(632.8\ \mathrm{nm}) = 3.3$, and $n_{\mathrm{a-Si_3N_4}}(632.8\ \mathrm{nm}) = 1.9$ denoting the measured refractive index, the index for a-Si:H, and for the stoichiometric SiN, respectively.[22]

Reference 12

The Sellmeier equation obtained by fitting the ellipsometry results has been given as

$$n_{\mathrm{PECVD}} = \sqrt{1 + \frac{2.503\lambda^2}{\lambda^2 - 17.29 \cdot 10^{-15}}} \qquad \text{(S5)}$$

Hence, $n_{\mathrm{PECVD}}(643\ \mathrm{nm}) = 1.9006$.

Reference 17

It is reported a value of $n_{\mathrm{PECVD}}(633\ \mathrm{nm}) = 1.96$.

SiN Urbach energy: LPCVD vs PECVD

The Urbach energies β^{-1} discussed in the main text are for LPCVD SiN only. Fig. S4 shows a comparison with PECVD films (diamond). These films present higher values of β^{-1} than LPCVD ones for similar Si/N values. This means that absorption due to electronic transitions between disorder-induced localized to extended states is increased in the former. Overall, β^{-1} reduces with Si/N for both deposition methods.

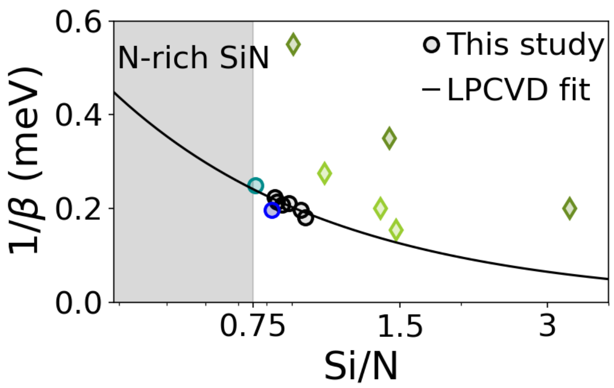

Figure S4: Urbach energy β^{-1} as a function of the Si/N ratio, for LPCVD (circles) and PECVD (diamond) SiN films. The compilation of data includes: blue, Ref. 10; darkcyan, Ref. 23; dark green, Ref. 24; light green, Ref. 25. The solid curve is a fitting curve of the form $f(x) = ae^b$ for the LPCVD data only, with $a = 0.1843$ meV and $b = -0.9427$.

XPS measurements

In this section, a more detailed overview is given regarding the XPS characterization. All measurements were carried out on a PHI Versa Probe III-spectrometer equipped with a monochromatic Al-Kα X-ray source and a hemispherical analyser (acceptance angle: $\pm 20°$). Pass energies of 140 eV and as well as step widths of 0.5 eV were used for survey and detail spectra, respectively. (Excitation energy: 1486.6 eV Beam energy and spot size: 50 W onto 200 μm; Mean electron take-off angle: 45° to sample surface normal; Base pressure: $< 8 \cdot 10^{-10}$ mbar, Pressure during measurements: $< 1 \cdot 10^{-8}$ mbar). Samples were mounted on double -sided polymer tape. Electronic and ionic charge compensation was used for all measurements (automatized as provided by PHI). The binding energy (BE) scale and intensity were calibrated by using methods described in ISO15472, ISO21270 and ISO24237. Surface cleaning was carried out using an Ar ion gun (2 kV, 2x2 mm, 1.3 μA, 5 min). Data analysis was performed using CASA XPS and Multipak software packages, employing transmission corrections, Shirley/Tougaard backgrounds[26,27] and customised Wagner sensitivity factors.[28]

References

(1) Cataldo, G.; Beall, J. A.; Cho, H.-M.; McAndrew, B.; Niemack, M. D.; Wollack, E. J. Infrared dielectric properties of low-stress silicon nitride. *Opt. Lett.* **2012**, *37*, 4200–4202.

(2) Macleod, H. A. *Thin-Film Optical Filters*; CRC Press, 2010.

(3) Zhang, C.; Giroux, M.; Nour, T. A.; St-Gelais, R. Radiative Heat Transfer in Freestanding Silicon Nitride Membranes. *Phys. Rev. Appl.* **2020**, *14*, 024072.

(4) Edalatpour, S.; Francoeur, M. Size effect on the emissivity of thin films. *J. Quant. Spectrosc. Raiat. Transf.* **2013**, *118*, 75–85.

(5) Bubenzer, A.; Koidl, P. Exact expressions for calculating thin-film absorption coefficients from laser calorimetric data. *Appl. Opt.* **1984**, *23*, 2886–2891.

(6) Land, A. T.; Dey Chowdhury, M.; Agrawal, A. R.; Wilson, D. J. Sub-ppm

Nanomechanical Absorption Spectroscopy of Silicon Nitride. *Nano Lett.* **2024**, *24*, 7578–7583, PMID: 38742810.

(7) Luke, K.; Okawachi, Y.; Lamont, M. R.; Gaeta, A. L.; Lipson, M. Broadband mid-infrared frequency comb generation in a Si3N4 microresonator. *Conf. Lasers Electro Opt. Eur. Tech. Dig.* **2015**, *2015-August*.

(8) Inukai, T. I. T.; Ono, K. O. K. Optical Characteristics of Amorphous Silicon Nitride Thin Films Prepared by Electron Cyclotron Resonance Plasma Chemical Vapor Deposition. *Jpn. J. Appl. Phys.* **1994**, *33*, 2593.

(9) Bulla, D.; Borges, B.; Romero, M.; Morimoto, N.; Neto, L.; Cortes, A. Design and fabrication of SiO/sub 2//Si/sub 3/N/sub 4/ CVD optical waveguides. 1999 SBMO/IEEE MTT-S International Microwave and Optoelectronics Conference. 1999; pp 454–457 vol. 2.

(10) Corato-Zanarella, M.; Ji, X.; Mohanty, A.; Lipson, M. Absorption and scattering limits of silicon nitride integrated photonics in the visible spectrum. *Opt. Express* **2024**, *32*, 5718.

(11) Worhoff, K.; Klein, E.; Hussein, G.; Driessen, A. Silicon oxynitride based photonics. 2008 10th Anniversary International Conference on Transparent Optical Networks. 2008; pp 266–269.

(12) Sorace-Agaskar, C.; Kharas, D.; Yegnanarayanan, S.; Maxson, R. T.; West, G. N.; Loh, W.; Bramhavar, S.; Ram, R. J.; Chiaverini, J.; Sage, J.; Juodawlkis, P. Versatile Silicon Nitride and Alumina Integrated Photonic Platforms for the Ultraviolet to Short-Wave Infrared. *IEEE J. Sel. Top. Quantum Electron.* **2019**, *25*, 1–15.

(13) Blasco-Solvas, M.; Fernández-Vior, B.; Sabek, J.; Fernández-Gavela, A.; Domínguez-Bucio, T.; Gardes, F. Y.; Domínguez-Horna, C.; Faneca, J. Silicon Nitride Building Blocks in the Visible Range of the Spectrum. *J. Light. Technol.* **2024**, 1–10.

(14) Lelit, M.; Słowikowski, M.; Filipiak, M.; Juchniewicz, M.; Stonio, B.; Michalak, B.; Pavłov, K.; Myśliwiec, M.; Wiśniewski, P.; Kaźmierczak, A.; Anders, K.; Stopiński, S.; Beck, R. B.; Piramidowicz, R. Passive Photonic Integrated Circuits Elements Fabricated on a Silicon Nitride Platform. *Materials* **2022**, *15*, 1398.

(15) Smith, J. A.; Francis, H.; Navickaite, G.; Strain, M. J. SiN foundry platform for high performance visible light integrated photonics. *Opt. Mater. Express* **2023**, *13*, 458.

(16) Mashayekh, A. T.; Klos, T.; Geuzebroek, D.; Klein, E.; Veenstra, T.; Büscher, M.; Merget, F.; Leisching, P.; Witzens, J. Silicon nitride PIC-based multi-color laser engines for life science applications. *Opt. Express* **2021**, *29*, 8635.

(17) Gorin, A.; Jaouad, A.; Grondin, E.; Aimez, V.; Charette, P. Fabrication of silicon nitride waveguides for visible-light using PECVD: a study of the effect of plasma frequency on optical properties. *Opt. Express* **2008**, *16*, 13509–13516.

(18) Sacher, W. D.; Luo, X.; Yang, Y.; Chen, F.-D.; Lordello, T.; Mak, J. C. C.; Liu, X.; Hu, T.; Xue, T.; Lo, P. G.-Q.; Roukes, M. L.; Poon, J. K. S. Visible-light silicon nitride waveguide devices and implantable neurophotonic probes on thinned 200 mm silicon wafers. *Opt. Express* **2019**, *27*, 37400.

(19) Poenar, D. P.; Wolffenbuttel, R. F. Optical properties of thin-film silicon-compatible materials. *Appl. Opt.* **1997**, *36*, 5122.

(20) Bonneville, D. B.; Miller, J. W.; Smyth, C.; Mascher, P.; Bradley, J. D.

Low-temperature and low-pressure silicon nitride deposition by ecr-pecvd for optical waveguides. *Appl. Sci. (Switzerland)* **2021**, *11*, 2110.

(21) Makino, T. Composition and Structure Control by Source Gas Ratio in LPCVD SiNx. *J. Electrochem. Soc.* **1983**, *130*, 450.

(22) MäcKel, H.; Lüdemann, R. Detailed study of the composition of hydrogenated SiN x layers for high-quality silicon surface passivation. *J. Appl. Phys.* **2002**, *92*, 2602–2609.

(23) Bauer, J. Optical properties, band gap, and surface roughness of Si3N4. *Phys. Status Solidi A* **1977**, *39*, 411–418.

(24) Garcia, S.; Bravo, D.; Fernandez, M.; Martil, I.; López, F. J. Role of oxygen on the dangling bond configuration of low oxygen content SiNx:H films deposited at room temperature. *Appl. Phys. Lett.* **1995**, *67*, 3263.

(25) Kato, H.; Kashio, N.; Ohki, Y.; Seol, K. S.; Noma, T. Band-tail photoluminescence in hydrogenated amorphous silicon oxynitride and silicon nitride films. *J. Appl. Phys.* **2003**, *93*, 239–244.

(26) Shirley, D. A. High-Resolution X-Ray Photoemission Spectrum of the Valence Bands of Gold. *Phys. Rev. B* **1972**, *5*, 4709–4714.

(27) Tougaard, S. Universality Classes of Inelastic Electron Scattering Cross-sections. *Surf. Interface Anal.* **1997**, *25*, 137–154.

(28) Wagner, C. Sensitivity factors for XPS analysis of surface atoms. *J. Electron Spectr. Rel. Phenom.* **1983**, *32*, 99–102.
